# Reminiscences of Dr. J. Marion Sims in Paris

**Published:** 1895-02

**Authors:** Edmond Souchon

**Affiliations:** Professor of Anatomy and Clinical Surgery, Tulane University, New Orleans, Louisiana


### Abstracts and Selections.

### Reminiscences of Dr. J. Marion Sims in Paris.

BY EDMOND SOUCHON, M. D.,
Professor of Anatomy and Clinical Surgery, Tulane University, New Orleans, Louisiana.

[Read at the meeting of the Southern Surgical Association, at Charleston, S. C., November 14, 1894.]

I have often related to my frinds the manner in which I happened to meet our surgical genius, Dr. J. Marion Sims, in Paris, and his first experiences in the French capital. They all were much interested in this, as they were in everything pertaining to this great and good man, and they repeatedly asked me to write out the little story for the benefit of the profession at large. I, to-day, comply with this wish, regretting very deeply for all concerned that my pen is not more gifted, so as to do better justice to my hero and to my readers.

In the fall of 1860, I entered the old Charity Hospital, on the rue Jacob, as a benevolent student, in the service of the venerable and world-renowned Professor Velpeau. It was my first beginning in the study of medicine. Facilities to beginners over there are not so great as they are here, in our Southern hospitals especially, and in order to have a chance to come in close contact with the professor and the patients, a simple voluntary student of the service had to be over-zealous with all, particularly the all-powerful interns or lords of the service. After several months of trial, I had succeeded in ingratiating myself with them all, and having been intrusted with the registration-book of the in-coming and out-going patients, with the number of bed, diagnosis, and dates, I had managed to to be on as fairly good terms with Professor Velpeau as a little insignificant nobody like myself could be with such a magnate as was the great Velpeau in such a place as the great Paris at that period. This may seem rather hard to American students, but it is all true, none the less.

Early one morning in the latter part of October, 1861, the year the War of Secession broke out, I was going to the hospital, and, as I was about to enter the gate, my attention was attracted at once by the face and appearance of a man who was coming toward the gate also, but from the opposite direction. That the face and appearance struck me at once, will readily be believed by all those who have had the happiness of knowing our great American surgeon. Its characteristic soft and sweet expression, together with his deep-set, bright eyes and prominent, bushy eyebrows, the half-smiling expression of his mouth, left uncovered by the absence of mustache or beard, made a much deeper impression on me than a glance ordinarily produces. I also at once recognized that he was a foreigner, and no Englishman at that, but surely and unmistakably an American, perhaps, hastily thought my young rebel heart, a Southerner; he must be that, I thought immediately afterward, because he looked so gentle and good, and yet, withal, so firm and self-reliant. All this took but a few seconds, and I continued my course toward the ward to which I was assigned, walking through the yard with another student, and the stranger coming up behind. When I reached the door of my ward, I went through and closed it; it was soon opened again, and closed; turning around, I noticed my "American." The doctor told me later that at the foot of the stairs the other student went in another direction than I, and he was perplexed for a moment as to which one he would follow; after a little hesitation, he said to himself: "Well, I think I will stick to the little one." The little one was myself.

I walked to the bed of the patient who took care of the white aprons the students wear over there, to protect their clothes, and as I was putting it on, the stranger came up to me, and after a most suave bow, said, in a very slow and deliberate manner, that the Frenchman he thought he was addressing might have some chance of understanding: "Will—Professor—Velpeau—be—here—to-day?" I burst out laughing, and answered him in fluent English: "No, sir; Professor Velpeau is absent on his vacation, and will not return before two weeks." The beautiful face brightened up at the sound of the English language where and when he so little expected it. "Where are you from," said he, "that you speak English?" "I am from the South, from New Orleans," said I, thinking that that would cool his enthusiasm if he were a Yankee. But far from it; he grasped my hand and pressed it so as to crush it. "Why," said he, "I also am a Southerner; I lived a long time in Montgomery, Alabama." That made us friends at once. I showed the doctor around the hospital, and finally asked him what he had come over here for.

"Well," said he, "I am Dr. Marion Sims, now living in New

York, who has invented a method of operating for vesico-vaginal fistula with almost invariable success.'' He looked at me to see if the name had made any impression on me, but it fell flat; we had never heard of Dr. Marion Sims in Paris. Furthermore, we all knew that nobody in the world knew anything about vesico-vaginal fistula except Professor Jobert de Lamballe, of the Hotel Dieu, and even under him, vesico-vaginal fistula was cured only exceptionally, even when using his procede de glissement (sliding process), and the idea of this new man coming to Paris to teach French surgeons how to cure vesico-vaginal fistula almost infallibly, somewhat shook my faith in my new friend.

He said he had a letter for Professor Velpeau, from Dr. Valentine Mott, of New York; that he was anxious to see the professor, to get a case to operate on before him, and thus to demonstrate his method. ''Well,'' I said, ''the professor will be here in some fifteen days," soon enough for your good, I thought to myself.

During that time, the doctor was living in a little boarding house in the rue de l'Universite, close to the hospital. He had invited me to dinner upon our very first meeting, and I went there once or twice to give him all the points about those men he was most likely to meet. He was all the time sanguinely confident, and looked so sweet, so modest, so magnetic, that I began to feel a very strong drawing toward him, and by the time Velpeau was to return, I was wound up to a high pitch, and as eager as my friend that he should have a case soon.

Finally Professor Velpeau arrived, and I sent word to Dr. Sims immediately; he soon came to present his letter, which Velpeau read at once and fluently, but when it came to speaking to Dr. Sims, he was at a loss to express himself, and looked around for me. It was always the case when prominent English visitors came to the hospital. Although the Englishmen read and wrote French as well as the Frenchmen read and wrote English, a good deal better than I could then, yet when it came to talking they had the greatest difficulty in understanding each other, and I was always hunted up to help them along. At that time Velpeau was nearing the end of a most hard-worked career, and, although age and success had softened him, yet he still bore the effects of his lowly, rough, peasant birth, and of his hard-earned victory. At times, the stiff haughtiness of the former years of struggle came over him. It did so, to some extent, at that time, and he was not to Sims exactly what he should have been, as I

thought, and his coldness to him made my heart ache somewhat, as I took that to be of ill omen. The truth was that the name American, at that time in Paris, always evoked the name of Barnum, and the fact that a comparatively young surgeon posed as a successful operator on vesico-vaginal fistula, when scarcely any one, not even Jobert de Lamballe, ever succeeded in Paris, had rather prejudiced Velpeau against Sims.

After a few, very few words, Velpeau said to me, "Eh bien, que veut-il?" "Well, what does he want?" I translated the sentence to Sims, who at once modestly but firmly answered, "I want a case to demonstrate my operation, if the professor will be kind enough to procure one for me." "All right," said Velpeau, "I will get him one," in a way that showed he had but little confidence in the final result. Then he turned around, without a hand shake or a word more to Sims, and went on with his visit. I felt quite hurt, but could say, and said, nothing. Velpeau was the idol of all young aspiring surgeons, the demi-god of the day. Nelaton was just looming up then, and had not as yet had his famous Garibaldi case, which gave him world-wide fame.

For several days, no case turned up. Sims was there every morning. I would introduce him to the younger surgeons who always swarmed around the old master, that he might not forget them. To every one who inquired about his object, he said, "I want a case," and I myself would then say, "He wants a case."

At last the case came! And, just as luck would have it, a case of moderate difficulty. "Thank the Lord," said I, with my American pride roused now to a high pitch for fear Sims should fail! When he told me he was sure to succeed, it was a great relief.

By this time the whole of the old Quartier Latin had heard of the news, which had rapidly spread from hospital to hospital. On the day of the operation, the famous little operating-theatre in the old Charity Hospital was overcrowded with students, and the arena below crowded also with the most distinguished professors of surgery of the French capital; Velpeau, Nelaton, Ricord, Malgaigne, etc., all but Jobert de Lamballe, who would not come.

Before beginning the operation, Dr. Sims proceeded to demonstrate it graphically, by using a piece of thick and hard cotton batting, through which he cut a hole representing the fistula, then he pared the edges slantingly in one strip, next he passed the silk threads and the wires, etc. The doctor called on me to translate as he spoke. I was not expecting this, and before such an audience, and I felt shy and scared, but he so insisted with his sweet eyes and smile that I got up, trembling all over, and with a quivering voice would repeat in French each sentence as he uttered it in English. Gradually, however, I found that it was not so very hard, I became emboldened and went through the whole procedure with comparative comfort. After the demonstration, Dr. Sims proceeded with the operation, which he performed with the skill and grace which characterized him. It was done in comparatively no time, closely watched and followed all the time by the French professors. When the doctor finally said it was done, a salvo of applause broke out from the benches; the professors rendered justice to the manner in which the operation had been performed, while reserving themselves mentally until the day when the sutures would be removed.

Dr. Sims attended to that case himself in the ward, and during the following days felt all along confident that it would be a success; and a success, a tremendous success, it turned out to be.

On the ninth day the same amphitheatre was again packed to witness the removal of the sutures; the case was pronounced cured, and this was confirmed by the French surgeons, who congratulated Dr. Sims.

The enthusiasm of the French students far exceeded their former outburst, and, since they could not very well carry Dr. Sims on their shoulders in triumph, they took hold of me in his place, and the resident students carried me to their mess-room to breakfast with them; a great and unprecedented honor in those days, for I was but a vulgar, simple, insignificant first-year student! I did more talking than eating, and the result of the excitement of mind and of heart was a very fine first-class headache that sent me to bed and lasted twenty-four hours.

However, some seemed to think that it might have been a chance cure. But these were soon to be set right. Immediately after the success of the first case, Dr. Sims started on the war path for another, which was soon procured for him by a physician in private practice. But this case came very near being a Waterloo. It was taken to a private place, the Hotel Voltaire, on the Quai Voltaire. The patient was a short, fat, stumpy little woman, and very obstinate. She, all of a sudden, absolutely refused to be operated upon unless she was given chloroform and put fast asleep. This much annoyed Dr. Sims, because in those days it was not thought quite safe to place a patient on the left side, the side of the heart, to give chloroform to the extent of keeping her perfectly still, since nothing could be done otherwise, and for such a long period as an hour or two. We were far then from the ideas of the present day. There was no overcoming her stubbornness and her will had to be done. It all went well for a while, a good while, but, all at once, the breathing became stertorous, the face blue, and the pulse flagged. The operation had to be suspended until she recovered. The operation was then resumed, but soon had to be stopped again, for the same reasons. Things were looking a little blue also, and as though the operator would not be able to complete the operation. But it was not to be so; it was to be completed, but it took Sims' whole nerve and skill to bring it to completion. During all that time the distinguished guests present said and did nothing, leaving Sims and his assistants to do all the fighting and get all the odium in case of failure, but all the credit in case of success. At one time I spoke to Velpeau to ask him what he thought of the condition of the patient, he shook his old silvery head, and I imparted to Sims what I took that to mean, that he might make the best of it. Finally the patient rallied and was put to bed. At the end of the usual time this case was pronounced a success.

A couple of weeks later Professor Jarjavay secured another case, upon which Dr. Sims operated at the Hospital St. Antoine. At the time of the removal of the sutures, a week or so later, Dr. Sims was not pleased with the appearance of the parts, and expressed his apprehension of some ulceration setting in and destroying the work done to a greater or lesser extent. Professor Jarjavay said that even if there was a fistulette (a small fistula) left, it would not matter much. Although he seemed to say this in a good spirit, yet it occurred to some that some people would be glad to hail this as a failure of the so-called infallible American method. But no fistulette occurred, and that case was also placed on record as a complete success.

From that time on cases were quite frequent, and naturally so, since hardly any were ever cured before, the stock of fistulæ was very great, and cases were not wanting. It was especially in private practice that they appeared to be abundant, and Dr. Sims scored success after success with the greatest ease. However, he again struck another hard case, even a worse one than the one at the Hotel Voltaire; it was the famous case of the "Countess" out in the country, in a chateau, a patient of Professor Nélaton. She also insisted upon taking chloroform, and when the operation was about half through, she showed all at once most alarming symptoms. It was then that Nèlaton uttered the legendary cry, "Head down," which every one conversant with these matters must remember. After much labor and still more anxiety the poor illustrious patient was revived and the operation was safely completed. It turned out also a complete success.

Cases followed one another wherever the doctor went; in Paris, London, Germany, etc., he was kept busy with fistulæ and other female cases. It was he who then sowed the first seeds of true gynecological science and art throughout Europe, the science so eminently and thoroughly American.

The doctor's reputation had acquired such proportions that the French Government presented him with the decoration of Knight of the Legion of Honor, the highest ambition of all Frenchmen.

—*Reprint.*

